# Supplementary material for: What works, for whom, and under what circumstances for recipients of training in opportunistic behaviour change conversations: A mixed methods realist evaluation protocol
Source: PLoS One. 2026 Aug 24;21(8):e0346348. doi: 10.1371/journal.pone.0346348 (PMC13502541; doi:10.1371/journal.pone.0346348)
Supplement: Supplementary file 1 [file pone.0346348.s001.docx]

Supplementary Material 1: Overall visual initial programme theory for VCSE service providers.
