# Supplementary material for: What works, for whom, and under what circumstances for recipients of training in opportunistic behaviour change conversations: A mixed methods realist evaluation protocol
Source: PLoS One. 2026 Aug 24;21(8):e0346348. doi: 10.1371/journal.pone.0346348 (PMC13502541; doi:10.1371/journal.pone.0346348)
Supplement: Supplementary file 3 [file pone.0346348.s003.docx]

Supplementary Material 3: Initial programme theories after literature scoping, stakeholder involvement, and prioritisation.

| **Theme** | | |
| --- | --- | --- |
| **Title** | **CMO configuration** | **Rival theory** |
| **Engagement (attending)** | | |
| Organisational support (attend) | If trainees aren’t mandated to attend, then their organisation needs to show support of their attendance (i.e. if they endorse it and are enthusiastic about it), and the content and intended outcomes of HCS training needs to be clearly communicated, so that trainees can feel justified in prioritising attendance to the training over their other roles and responsibilities. | If trainees are not encouraged or mandated by leadership and management at their organisation to attend HCS training, then they will likely not attend because it is not prioritised, and trainees would therefore feel a sense of guilt about spending work time attending HCS training. |
| **Engagement (attention)** | | |
| Personal interest | If trainees are mandated to attend (e.g. pharmacy students), then HCS training needs to be framed around trainees’ personal interests (e.g. to achieve core competencies, to help them communicate on placement) for trainees to engage in the training, because they are not intrinsically motivated to attend and instead require extrinsic motivation and promise of personal reward. Engagement in the training will mean they are more likely to change their belief about what works, improve their HCS skills, and ultimately implement HCS into their existing conversations. |  |
| **Acquisition during training** | | |
| Mindset shift | If trainees (coming from diverse professional background) are experienced in having health and wellbeing conversations (e.g. VCSE service providers) and are open to changing their approach to conversations in order to help service users in any way they can, then the experiential learning aspect of Healthy Conversation Skills training will encourage them to contrast their existing way of approaching conversations with Healthy Conversation Skills, helping them to learn the new skills and undergo a mindset shift in their approach. This will make them more likely to approach conversations with their newly learned Healthy Conversation Skills- such as applying open discovery questions- and mean that they spend more time telling/suggesting during healthy conversations with service users. | If trainees have a limited perception of the scope of their ability to help service users within their role, then they will be less likely to be willing to attend HCS training. If they are encouraged to by their organisation, then they will likely disengage from the active learning of the skills because they will not feel that HCS training is relevant for them. This will mean that they either do not attend HCS training at all, or that they will attend but not acquire the skills needed to deliver HCS.  If trainees currently do not engage in conversations with service users (e.g. pharmacy students), then HCS the content on HCS is likely to feel too overwhelming and advanced for their level of communication skills, and they will be unable to draw upon any previous experiences of conversations and amend their approach. This could result in frustration and confusion, and the skills are unlikely to be applied. |
| Proactive conversations | For individuals who default to active listening and providing empathy during conversations about health and wellbeing (e.g. VCSE service providers), the content within HCS about facilitating SMARTER goal setting will encourage trainees to see value in health and wellbeing conversations having a proactive nature. This will make them more likely to facilitate conversations that reach a goal or agreement (e.g. to go and visit a specific site or service), although this may not necessarily be through SMARTER goal setting. |  |
| Holistic health and wellbeing | If trainees are motivated to help in any way that they can and are experienced in having conversations with service suers (e.g. VCSE service providers), then the HCS training which is framed by this motivation and portrays health and wellbeing in a general sense will prompt trainees to view health and wellbeing in a holistic way rather than limited to what they may have previously considered as out of the scope of their role. This will widen the scope of how trainees perceive they can help people improve their health and wellbeing, meaning they will be more likely to feel able to encourage positive behaviour change relating to the topic that is most important to the individual. | If trainees are not experienced in communicating with service users (e.g. pharmacy students), then HCS training that is framed holistically will feel overwhelming and irrelevant to the trainee’s role, and trainees will see limited applicability to their practice and disengage from the training. |
| SMARTER goal facilitation | If trainees are experienced in having health and wellbeing conversations and work in a role where they often have structured conversations (i.e following a specific procedure) that provides a place (e.g. a case file, virtual or physical) to record and monitor conversations and they have repeat interactions with the same person, then they will plan to apply the training content and resource on SMARTER goal setting when prompted to consider what element of the training to implement post training, because it is perceived to fit within their existing conversations. Thus, when they return to work following the training, they will have an opportunity to facilitate and monitor SMARTER goal setting, which will help recipients implement a behaviour change. If it is received positively, this will become a habitual part of the existing structured conversations that trainees have within their role. | Even if a trainee has structured and repeated conversations with an opportunity to record and monitor them, they may not apply SMATER goal setting if they prefer an informal and open approach rather than a structured approach. This will mean that they plan to use other HCS skills when prompted to select one thing that they will implement, meaning they miss an opportunity to facilitate SMARTER goal setting.  If a trainee does not have repeated structured conversations with opportunities to refer to the SMARTER framework and monitor it, they will likely forget the content on SMARTER goals quickly even if they felt an affinity towards it, because they are unable to imagine how they could implement it in their everyday practice. They will therefore not facilitate SMARTER goal setting, although they may feel inspired to apply a proactivity to conversations (see ‘proactive conversations above), such as by asking the individual what they plan to do next. |
| Critical reflection | If individuals are confident enough in their own abilities to be able to critique their existing communication style that they have during existing interactions with service users, then the content of the HCS approach combined with opportunities to practice conversations throughout the training will help them to critically reflect on their existing practice, helping them to change their beliefs around what an effective conversation is. This will increase their motivation to apply the HCS approach instead of the approach they previously adopted (e.g. advice giving, providing empathy only, deferring to signposting), so that they intend on applying it after the training. | If a trainee is not able to critically reflect on whether their existing practice could be improved, then the opportunity to reflect may cause them to feel defensive about their existing communication approach, such that the training causes them to ‘double down’ on what they believe to be their existing favourable communication style. This will mean that they have no motivation and therefore intention of applying their HCS training after the training. |
| Planning and intentions | If trainees have a role where the conversations that they have are generally predictable (i.e. they often follow a specific event) and/or structured (e.g. they follow a structured needs assessment), then when they are prompted to think about when they will put their HCS training into practice, they will be able to provide a detailed plan of when they intend to implement the training, which is attached to a specific cue. This will make them more likely to implement HCS after the training, and if they find that it enhances their existing conversations with service users, they are more likely to adopt HCS as habit, even outside of the structured and predictable conversation that they initially tried HCS. | If trainees have a varied role where they have no regularity of when service users talk about wellbeing, particularly if they are not surrounded by other who have also completed HCS training, then they will be unlikely to be able to form a specific plan about when they will implement HCS when prompted about what they will take away from the training. This will mean that they are unlikely to practice HCS immediately after the training, and the skills may be quickly forgotten. |
| Provision of examples | For trainees who are likely not yet confident in having health and wellbeing conversations with service users (e.g. pharmacy students), bespoke training is optimal because the specific examples from practice that is relevant/ similar to theirs helps them to see when HCS could be applied, which helps them to contextualise the skills and makes the content feels relevant and therefore memorable, making them more likely to learn them. This will mean that they will be more likely to adopt the HCS approach during future conversations at work or on placement. | If trainees not experienced in health and wellbeing conversations (e.g. pharmacy students) are not provided with bespoke training that provides specific examples relevant to their practice, then the content around the HCS philosophy and skills will feel abstract and intangible. The trainee will have no reference point for which to ‘hang’ their newfound knowledge and skills upon, so they will likely be quickly forgotten and not applied within future conversations. |
| **Applying the skills** | | |
| Reinforcement of previous training | If trainees are not yet experienced in having health and wellbeing conversations (e.g. pharmacy students), and the skills taught in the HCS are similar or complement existing training that trainees have received, then the content on the skills (including practicing them) will be remembered because it has reinforced existing learning in a different context, thereby making it more salient. This will be sufficient to increase trainee’s confidence to have conversations with service users including about heath and wellbeing, but insufficient to implement the specific skills (e.g. ODQs, SMARTER goal setting). | If trainees are experienced in having health and wellbeing conversations (e.g. VCSE service providers), and the skills taught in the HCS are similar or complement existing training that trainees have received, then the content on the skills will merely reinforce trainee’s confidence in a person-centred and motivational approach, and will therefore have a minimal effect on their behaviour during health and wellbeing conversations. |
| Organisational support (implement) | If trainees are encouraged by leadership and management at their organisation (i.e. if they endorse it and are enthusiastic about it) to attend and adopt the HCS approach, then trainees will feel confident to implement the skills they learn during the training because they will feel that the HCS approach is a social norm and therefore is socially accepted. This will make it more likely for individuals to adopt HCS as their communication style on a regular basis, leading to habit formation. | If trainees are not supported by leadership and management at their organisation to adopt the HCS approach but decide to self-enrol on the training because they feel passionate and proactive about HCS, then they are unlikely to adopt the skills long-term because they will feel that the HCS approach is not socially accepted which will be a barrier to them using the skills. |
| Cues and reminders | If trainees learn the HCS skills and philosophy during HCS training and are motivated to use it, then behavioural cues in their practice environment (e.g. situations that remind trainees of examples from the training, a HCS badge, a HCS mouse mat, even the follow-up confidence and competence survey as part of this evaluation) will remind them to apply the HCS approach. Continued cues will help trainees to embed the approach into habit. | Even if trainees leave feeling motivated and able to adopt the HCS approach, they might forget to change their existing communication style if they are not reminded of the training. |
| **Habit formation** | | |
| Reactions from recipients | If trainees who have completed HCS training go on to deliver the HCS approach and receive a positive response from recipients (e.g. they become enthused or provide positive feedback), then trainees will feel a great sense of reward and agency at helping other people. This will motivate them to continue applying the HCS approach, bolstered by their self-belief in their ability to support people to make a behaviour change, and therefore HCS will become habitual for them. | If trainees who have completed HCS training go on to deliver the HCS approach and receive a negative reaction from recipients, trainees will feel awkward or embarrassed or guilty. This will make it unlikely that they will continue to apply the HCS approach to avoid these uncomfortable emotions in themselves and recipients, and more likely that they will return to their default communication style. Thus, the HCS communication style will not become habitual. |
