# Supplementary material for: What works, for whom, and under what circumstances for recipients of training in opportunistic behaviour change conversations: A mixed methods realist evaluation protocol
Source: PLoS One. 2026 Aug 24;21(8):e0346348. doi: 10.1371/journal.pone.0346348 (PMC13502541; doi:10.1371/journal.pone.0346348)
Supplement: Supplementary file 4 [file pone.0346348.s005.docx]

**Response to reviewer comments**

| **Reviewer comments** | **Author response** |
| --- | --- |
| The introduction could be strengthened by more clearly situating MECC within the broader spectrum/framework of behaviour change interventions, of which it represents one component. While the protocol identifies MECC as an opportunistic brief or very brief intervention, further clarification of how this aligns with established categories of intervention intensity (e.g. very brief, brief, extended brief, and high-intensity interventions) would improve conceptual clarity.  This would help position MECC appropriately within the wider behaviour change framework and reduce potential misconceptions that it is intended to address behaviour change across all levels of need. Drawing on frameworks such as NICE guidance on individual-level behaviour change and the NHS England Behaviour Change Development Framework would strengthen this contextualisation within the rationale.  Providing this context would also enable the realist evaluation to contribute to understanding whether MECC HCS is most appropriately conceptualised as a very brief or brief intervention, or whether it aligns more closely with other levels of intervention intensity in practice. | Thank you for your thoughtful and incredibly useful comments. Your review has significantly improved our manuscript, particularly in framing the implications of the findings we will produce. See our responses to your individual comments below.  Thank you for this comment- I agree that this should be highlighted, to avoid over-promising the impact and application of MECC. I have therefore added the following section into the introduction of the manuscript:  ‘*Within the wider framework of individual-level behaviour change interventions, MECC is conceptualised as a low-intensity, opportunistic behaviour change intervention (5). NICE guidance on individual-level behaviour change (PH49) distinguishes between very brief, brief, extended brief, and high-intensity interventions, delivered according to an individual’s level of need (7, 8). MECC is typically categorised within the lower-intensity end of this continuum, spanning very brief and brief interventions (5). As such, MECC conversations may range from a matter of seconds to 30 minutes and support self-management, requiring signposting or referral to more specialist services where more complex or sustained support is required (5, 8). Thus, MECC is positioned as an entry-point within a broader behaviour change system.*’  And the following at the end of the introduction:  ‘*More broadly, understanding how HCS training shapes the delivery of MECC as situated as a brief, low intensity intervention is important for clarifying its intended function and mechanisms in practice.*’ |
| The focus on “what works and for whom” is clearly articulated. However, it would be helpful to explicitly clarify that this realist evaluation is focused on MECC HCS as a workforce intervention, specifically aiming to build confidence and competence among trainees.  As currently presented, there is potential for readers to assume that the study will assess downstream patient or population-level outcomes. It would strengthen the protocol to clearly state that evaluating behavioural outcomes among recipients of MECC conversations is beyond the scope of this study (if this is the case). If the authors have considered assessing any downstream behavioural impacts, even in a limited or exploratory way, it would be useful to outline this. If not, a clear statement of scope and limitations would help manage expectations and improve transparency. | Thank you for this comment- the last paragraph of the introduction has now been amended to provide clarity about what is and is not within scope of the current study:  ‘*The resulting programme theory will inform on how HCS training as a workforce intervention can be adapted for different audiences, to make sure that it equips all recipients with the knowledge, confidence, and motivation to deliver MECC. It is therefore noteworthy that evaluating behavioural outcomes of recipients of MECC conversations is beyond the scope of this study. Nonetheless, the findings will facilitate the future evaluation of MECC delivery in the VCSE by exploring whether HCS training is sufficient for MECC delivery*.’ |
| In addition, clarifying the scope in this way would also strengthen the study’s contribution by enabling the realist evaluation to more clearly position MECC HCS within the wider spectrum/framework of behaviour change interventions. This could help determine whether MECC HCS is most appropriately conceptualised as a very brief or brief intervention, or whether it aligns more closely with other levels of intervention intensity in practice. | Please see my additions to the discussion section described in box 1. |
| While the protocol clearly outlines outcomes related to confidence and competence following HCS training, it would be helpful to further clarify how these outcomes are expected to translate into actual delivery of MECC conversations in practice. At present, the link between increased capability (e.g. confidence and competence) and behavioural enactment (i.e. having MECC conversations) could be made more explicit within the programme theory and outcome framework. | Thank you for this comment. I am hesitant to amend the IPTs in any way, given that data collection has already commenced and the programme theories have already developed since starting data collection. However, I have amended the overall IPT to better represent the existing IPTs around translating knowledge, skills, and motivation into practice (see the edited section below). As shown in Figure 1, this is already an important part of the initial programme theories. I agree that this link was not made explicit in the original version of the overall IPT.  ‘*HCS ‘lite’ training helps people to deliver health behaviour change conversations because trainees exchange their existing communication style with a new proactive and motivational approach, through reflection and immersion in the skills. However, to translate intention to delivery after HCS ‘lite’ training, trainees must feel that the HCS approach is a social norm within their organisation and thus safe to deliver, or be prompted through environmental cues and reminders, which is particularly impactful if trainees have already planned to deliver HCS during the training*.’ |
| Relatedly, the relatively short follow-up period (approximately eight weeks post-training) may limit the ability to assess whether HCS is embedded into routine practice over time. It would be useful to acknowledge this as a limitation, particularly given that sustained behaviour change and habit formation are key to MECC implementation. | Thank you for this comment- this potential issue has been expanded upon and defended in the discussion section:  ‘*Similarly, whilst an eight-week follow-up period (selected as sufficient to demonstrate habit formation (58)) may limit conclusions that can be drawn about how HCS training is embedded over time, the interviews will inform this objective by including participants from a broad range of durations from receiving HCS training*.’ |
| Finally, given the range of terminology used (e.g. MECC, HCS, opportunistic behaviour change conversations), providing a clear and concise definition of these concepts early in the manuscript would improve clarity for readers, particularly those less familiar with this area. | Thank you for raising this- I have now added a table to explain key concepts relating to the topic of the study (see Table 1). |
